# RETRACTION: Enhancing Wound Healing and Overcoming Cisplatin Resistance in Ovarian Cancer

**DOI:** 10.1111/iwj.70483

**Published:** 2025-04-02

## Abstract

**RETRACTION:** ZhangQ. , GuoF. , LiuH. , and HongL. , “Enhancing Wound Healing and Overcoming Cisplatin Resistance in Ovarian Cancer,” International Wound Journal 21, no. 4 (2024): e14569, 10.1111/iwj.14569.38158767 PMC10961880

The above article, published online on 29 December 2023, in Wiley Online Library (http://onlinelibrary.wiley.com/), has been retracted by agreement between the journal Editor in Chief, Professor Keith Harding; and John Wiley & Sons Ltd. Following an investigation by the publisher, all parties have concluded that this article was accepted solely on the basis of a compromised peer review process. The editors have therefore decided to retract the article. The authors did not respond to our notice regarding the retraction.

**RETRACTION:** Q. Zhang , F. Guo , H. Liu , and L. Hong , “Enhancing Wound Healing and Overcoming Cisplatin Resistance in Ovarian Cancer,” International Wound Journal 21, no. 4 (2024): e14569, 10.1111/iwj.14569.38158767 PMC10961880

The above article, published online on 29 December 2023, in Wiley Online Library (http://onlinelibrary.wiley.com/), has been retracted by agreement between the journal Editor in Chief, Professor Keith Harding; and John Wiley & Sons Ltd. Following an investigation by the publisher, all parties have concluded that this article was accepted solely on the basis of a compromised peer review process. The editors have therefore decided to retract the article. The authors did not respond to our notice regarding the retraction.

